# Differences Between Medication-Related Osteonecrosis of the Jaw Caused by Bisphosphonates and Denosumab: Histological, Molecular Biological, and Clinical Studies

**DOI:** 10.7759/cureus.62855

**Published:** 2024-06-21

**Authors:** Taro Miyoshi, Mitsunobu Otsuru, Kota Morishita, Keisuke Omori, Kei-ichiro Miura, Saki Hayashida, Satoshi Rokutanda, Yuki Matsushita, Masahiro Umeda, Tomohiro Yamada

**Affiliations:** 1 Department of Oral and Maxillofacial Surgery, Nagasaki University Graduate School of Biomedical Sciences, Nagasaki, JPN; 2 Oral and Maxillofacial Surgery, Graduate School of Kanagawa Dental University, Kanagawa, JPN; 3 Oral and Maxillofacial Surgery, Nagasaki University, Nagasaki, JPN; 4 Dentistry and Oral Surgery, The Japanese Red Cross Nagasaki Genbaku Hospital, Nagasaki, JPN; 5 Dentistry and Oral and Maxillofacial Surgery, Juko Memorial Nagasaki Hospital, Nagasaki, JPN; 6 Cell Biology, Nagasaki University, Nagasaki, JPN

**Keywords:** bisphosphonates, bone resection, denosumab, mronj, osteonecrosis of the jaw, polymerase chain reaction

## Abstract

Purpose

Medication-related osteonecrosis of the jaw (MRONJ) is a serious side effect of antiresorptive agents such as bisphosphonates (BPs) and denosumab (DMB). We investigated whether a difference exists between BP- and DMB-related osteonecrosis of the jaw (ONJ).

Patients and methods

Histological images of 30 patients with BP-related ONJ and 13 patients with DMB-related ONJ were observed using hematoxylin-eosin and cathepsin K staining. Moreover, bone metabolism markers in the blood and bone mineral density were measured in 18 patients with BP-related ONJ and five patients with DMB-related ONJ. Furthermore, we conducted a quantitative analysis of local bone metabolism-related genes using surgical specimens through real-time reverse transcription polymerase chain reaction. Additionally, a retrospective study of 298 patients with MRONJ examined the differences in the characteristics of BP- and DMB-related ONJ and the factors associated with treatment outcomes.

Results

Histological examination revealed that patients treated with DMB had more severe osteoclast suppression than those treated with BP. No significant difference was observed in blood-bone metabolism markers between the two drugs; however, the suppression of local bone metabolism-related genes was stronger in patients treated with DMB. Clinical studies indicate that DMB-related ONJ is more frequently observed without osteolysis.

Conclusion

BP-associated ONJ and DMB-associated ONJ were shown to differ slightly. Clinical studies indicate that osteolysis is often unclear in DMB-related ONJ, and methods of bone resection during surgery need to be established.

## Introduction

Osteoporotic fractures or skeletal-related events due to bone metastases of malignant tumors significantly reduce the quality of life for patients. Antiresorptive agents (ARAs), such as bisphosphonate (BP) and denosumab (DMB), are frequently used to reduce the risk of these complications. Despite its potency in inhibiting bone resorption, ARA can cause medication-related osteonecrosis of the jaw (MRONJ) as a severe adverse event.

MRONJ was first reported by Marx in 2003 as an intractable form of osteonecrosis of the jaw (ONJ) in patients using BP, and approximately 20 years have passed since then [1]. In 2010, Taylor et al. reported a similar disease in patients using DMB, an anti-RANKL antibody [2]. To date, many cases have been reported, and several position papers and guidelines have been published [3-6].

BP and DMB are potent inhibitors of osteoclast function; however, their mechanisms are different [7-11]. BP is deposited in the bone for long periods and is incorporated into osteoclasts as they resorb bone, resulting in apoptosis. Influenced by BP, osteoclasts transform into large multinucleated cells that detach from the surface of the bone. On the other hand, DMB does not bind to bone, is incorporated into the serum, and circulates in the blood. The half-life of DMB is approximately 25 days. DMB inhibits osteoclast differentiation into mature osteoclasts and promotes osteoclast survival. As a result, the total number of osteoclasts is reduced under the influence of DMB, and small immature osteoclasts are observed. DMB is more effective than BP at suppressing skeletal-related events in patients with cancer [12], and the incidence of MRONJ is slightly higher with DMB than with BP [13]. However, no study has examined whether the pathophysiology, imaging findings, or resistance to treatment differs between BP- and DMB-related ONJ.

MRONJ is a refractory ONJ that occurs in patients receiving ARA and is often diagnosed based on bone exposure or a fistula that reaches the bone; however, latent cases with neither bone exposure nor a fistula are rare. In the American Association of Oral and Maxillofacial Surgeons (AAOMS) position paper (2022) [6], the imaging findings of stage 0 MRONJ are described as “alveolar bone resorption not attributable to chronic periodontal disease,” and osteolysis has been recognized as the earliest imaging finding of MRONJ. Moreover, one definition of stage 3 states that osteolysis extends to the inferior border of the mandible or the sinus floor. Osteolysis is a significant imaging finding in the diagnosis and staging of MRONJ and in determining treatment methods.

In addition to osteolysis, another clinically important imaging finding is a periosteal reaction [14]. Periosteal reactions are relatively frequent imaging findings in patients with MRONJ (18% [15], 21% [16], 21.3% [17], and 60% [14]), whereas they are rarely seen in patients with osteoradionecrosis (ORN) [14,15]. As for its clinical significance, Kojima et al. first reported in 2019 that the postoperative course of MRONJ with periosteal reaction was significantly worse [17]. In 2021, Soutome et al. reported that among periosteal reactions, the gap type, which shows a gap between the existing bone, and the irregular type, which shows irregular morphology, are infectious lesions by real-time polymerase chain reaction (PCR) analysis and that these types of periosteal reaction should be included in the resection area during surgery [16,18]. Kojima et al. reported that MRONJ with a periosteal reaction is prone to lesion enlargement during conservative treatment [19].

Osteosclerosis is another common imaging finding observed in patients with MRONJ. Osteosclerosis is often diffuse and extensive and has not always been included in the resection area during surgery. However, mixed-type osteosclerosis, which contains many small radiolucent areas within the osteosclerosis, has a significantly higher recurrence rate if left untreated at surgery. Real-time PCR has demonstrated the presence of numerous bacteria and fungi within the bone showing mixed-type osteosclerosis [20]. Thus, imaging findings such as osteolysis, periosteal reaction, and osteosclerosis are crucial for diagnosing and treating MRONJ.

Osteolysis and osteosclerosis are common radiographic findings in almost all cases of MRONJ, regardless of the stage. Moreover, sequestrum separation and periosteal reactions are often observed. On the other hand, atypical types of MRONJ have also been reported in recent years. Otsuru et al. reported a periosteal reaction dominant type showing periosteal reaction with or without osteolysis [21]. Recently, Sakamoto et al. [22], and Kojima et al. [23] reported MRONJ with no abnormal CT findings, such as osteolysis, sequestrum formation, or periosteal reaction. They stated this non-osteolytic MRONJ occurs in patients receiving high-dose DMB therapy. We hypothesized that DMB would be more osteoclast suppressive than BP and produce MRONJ without osteolysis. This study aimed to investigate the differences in histological findings, local bone metabolic markers, imaging findings, treatment, and prognosis between BP- and DMB-related ONJ.

## Materials and methods

Histological study

The participants were patients with osteoporosis and MRONJ who underwent surgery at the Department of Oral and Maxillofacial Surgery, Nagasaki University Hospital, between March 1, 2011, and December 30, 2020. The data were accessed from the medical records on February 20, 2021, for research purposes. Because few patients with malignant tumors were treated with BP and most were treated with DMB, histological investigation was conducted only in patients with osteoporosis.

A total of 43 patients were enrolled in the histological study. Thirty patients (12 males and 18 females) received BP therapy, eight patients (one male and seven females) received BP initially but were changed to DMB therapy, and five patients (three males and two females) received DMB therapy. The average age of the patients was 81.5 ± 8.81 years: 78.8 ± 7.35 in patients receiving BP, 85.3 ± 4.84 in those receiving BP initially but changed to DMB, and 72.7 ± 10.8 in those receiving DMB.

The morphology and number of osteoclasts were observed using hematoxylin-eosin and cathepsin K staining of specimens obtained from the resected margins. Cases in which obtaining a living bone tissue specimen was not possible because of the removal of the healthy bone surrounding the necrotic area or where the resection margins consisted solely of necrotic bone were excluded. Histopathology sections were decalcified in 0.5 M ethylenediaminetetraacetic acid, and embedded in paraffin to prepare 4-μm-thick sections. Antigen activation in immunostaining was performed in a target retrieval solution (DAKO, Santa Clara, CA) using the warm bath method. The primary antibody was rabbit anti-cathepsin K polyclonal antibody (1:2000, Abcam: ab19027), and the secondary antibodies used were EnVision+ System-HRP labeled polymer anti-rabbit (DAKO). For immunohistochemical reactions, the Liquid DAB+ Substrate Chromogen System (DAKO) was used, and Meyer’s hematoxylin was used for nuclear staining. Osteoclasts detached from the bone surface or small cathepsin K-positive cells with a small number of nuclei were found to be suppressed. Mild suppression was designated when only normal osteoclasts were present, moderate suppression when normal and suppressed osteoclasts coexisted, severe suppression when only suppressed osteoclasts were present, and no suppression when neither normal nor suppressed osteoclasts were detected.

The study protocol conformed to the ethical guidelines of the Declaration of Helsinki and the Ethical Guidelines for Medical and Health Research Involving Human Subjects by the Ministry of Health, Labor, and Welfare of Japan. Ethical approval was obtained from the Institutional Review Board (IRB) of Nagasaki University Hospital (#21021509). Identifiable patient information was removed because of the retrospective nature of the study, and the research protocol was posted on the Nagasaki University Hospital's website, along with an opt-out option, as directed by the IRB.

Serum bone metabolism markers, bone mineral density, and local bone metabolism markers

The participants were patients with MRONJ who underwent surgical treatment at the Department of Oral and Maxillofacial Surgery at Nagasaki University Hospital between September 2014 and August 2015. Only cases in which live bone tissue from the resection margins could be harvested were used. A total of 23 patients were enrolled in this study. Among them, BP was prescribed for three men and 15 women, with a mean age of 78.1 ± 8.6 years. Of these patients, 14 had osteoporosis, and four had malignant tumors. In contrast, DMB was administered to four men and one woman, with an average age of 68.6 ± 11.6 years. Among them, two patients had osteoporosis, and three had malignant tumors.

Serum was isolated from the harvested blood during preoperative testing. Serum calcium (Ca), tartrate-resistant acid phosphatase 5b (TRACP-5b), and bone alkaline phosphatase (BAP) were measured.

CT analysis of the head and neck region was performed to investigate the area of the lesion and determine the extent of resection. Regions of interest with a 0.597 cm^2^ area were set in the body of the third cervical vertebra, as previously described [24]. The CT values were averaged, and the mean CT value was transformed into the bone mineral density (BMD).

The resected specimen of viable bone around the sequestrum was frozen in liquid nitrogen and crushed into a powder using an SK Mill (Tokken, Chiba, Japan). Total ribonucleic acid (RNA) was extracted using TRIzol reagent (Invitrogen Life Technologies, Paisley, UK) and a total RNA isolation kit (Macherey-Nagel, Duren, Germany). For real-time reverse transcription polymerase chain reaction (RT-PCR), aliquots of 1 μg of RNA were reverse transcribed to complementary deoxyribonucleic acid using ReverTra Ace (Toyobo, Osaka, Japan). Expression was quantified by real-time PCR using a Stratagene System (Agilent Technologies, Santa Clara, CA). The relative amount of each messenger RNA (mRNA) was normalized to beta-actin (β-actin) expression. Primer sequences are listed in Table 1.

**Table 1:** Primer sequences used for reverse transcription polymerase chain reaction. Trap: tartrate-resistant acid phosphatase; Ctsk: cathepsin K; Dmp1: dentin matrix acidic phosphoprotein-1; Alp: alkaline phosphatase; Ocn: osteocalcin.

Gene	Forward primer (5'-3')	Reverse primer (5'-3')
Trap	GATCCTGGGTGCAGACTTCA	GCGCTTGGAGATCTTAGAGT
Ctsk	ACCGGGGTATTGACTCTGAA	GAGGTCAGGCTTGCATCAAT
Dmp1	CAGGAGCACAGGAAAAGGAG	GCATTGGTGTTGTACGTCTTG
Alp	CAACCCTGGGGAGGAGAC	ACCTTTGCTGGACTCTGCAC
Ocn	TGAGAGCCCTCACACTCCTC	ACCTTTGCTGACTCTGCAC
B-actin	AAACTGGAACGGTGAAGGTG	TCAAGTTGGGGGACAAAAAG

The study protocol conformed to the ethical guidelines of the Declaration of Helsinki and the Ethical Guidelines for Medical and Health Research Involving Human Subjects by the Ministry of Health, Labor, and Welfare of Japan. Ethical approval was obtained from the Ethics Committee of Nagasaki University Graduate School of Biomedical Sciences (#1505). Written informed consent was obtained from all patients who underwent molecular biological studies of local bone metabolism markers.

Clinical study

**Figure 1:**
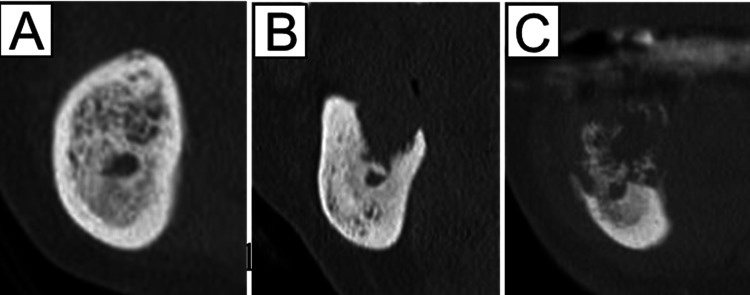
Degree of osteolysis. (A) No osteolysis, (B) localized osteolysis above the mandibular canal, and (C) extended osteolysis involving the mandibular canal.

**Figure 2:**
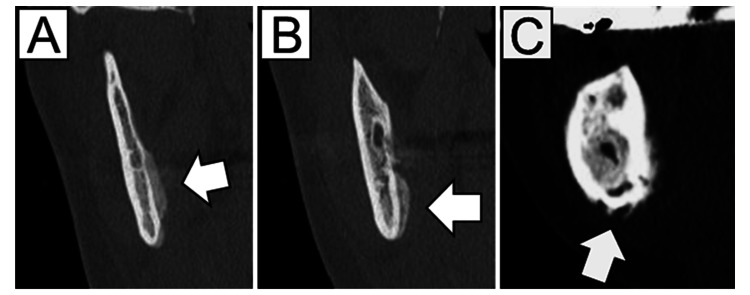
Periosteal reaction. (A) Attached-type, (B) gap-type, and (C) irregular-type.

**Figure 3:**
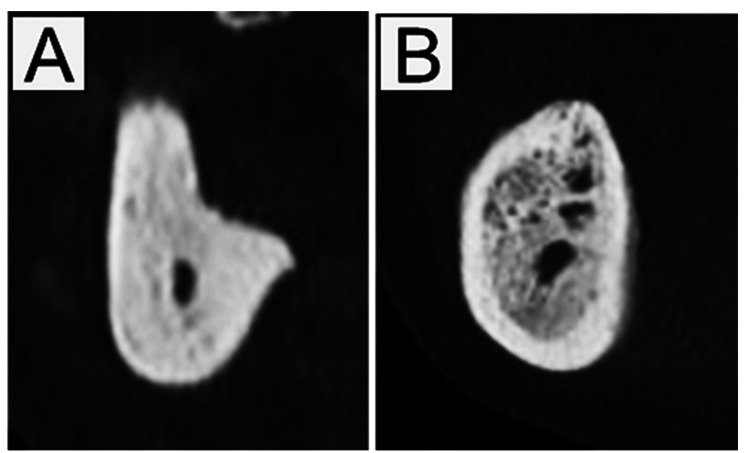
Osteosclerosis. (A) Uniform-type and (B) mixed-type.

The participants were 298 patients with MRONJ who underwent surgery at the Department of Oral and Maxillofacial Surgery of Nagasaki University Hospital between March 1, 2011, and December 31, 2022. The data were accessed from the medical records on August 30, 2023, for research purposes. From the medical records, we investigated age, sex, site (maxilla or mandible), MRONJ stage (AAOMS 2022), primary disease (osteoporosis or malignancy), body mass index, neutrophils, lymphocytes, neutrophil-to-lymphocyte ratio, serum albumin, ARA type (BP, DMB, initially BP and then switched to DMB), duration of ARA administration, drug holiday before surgery for more than two months, bone exposure, fistula reaching bone, number of surgeries, and treatment outcome (healing or non-healing). Radiographic findings were determined from CT images taken before surgery, including the degree of osteolysis (none, localized, or extended), periosteal reaction, and mixed osteosclerosis. The degree of osteolysis was defined as “localized” if the nasal cavity and maxillary sinus floor were preserved in the maxilla cases or if the mandibular canal was not included in the mandibular cases, “extended” if the nasal cavity and maxillary sinus floor were resorbed in the maxilla cases or if the mandibular canal was included in the mandibular cases, and “none” if no bone resorption was observed except for the remaining extraction socket (Figure 1). According to the classification of Soutome et al., periosteal reactions were classified as “none,” “attached-type,” “gap-type,” and “irregular-type” (Figure 2) [16,18]. Furthermore, we defined mixed-type osteosclerosis [20] as the presence of multiple small radiolucent areas within marked osteosclerosis (Figure 3). Treatment outcome was defined as “healing” if all symptoms, including bone exposure, disappeared and “non-healing” otherwise.

Univariate and multivariate Cox regression analyses were performed to determine the association between each factor and the treatment outcome. Kaplan-Meier curves were plotted for factors that were significant in the multivariate analysis. The differences between BP- and DMB-related ONJ were examined using Fisher’s exact test, one-way analysis of variance, or Mann-Whitney U test, and logistic regression analysis was performed on the factors that were significant in these tests. All statistical analyses were performed using SPSS version 26.0 software (Japan IBM Co., Ltd., Tokyo, Japan), and two-sided p-values less than 0.05 were judged statistically significant.

The protocol of this study complies with the ethical guidelines of the Declaration of Helsinki and the Ethical Guidelines for Medical and Health Research Involving Human Subjects by the Ministry of Health, Labor, and Welfare of Japan. Ethical approval was obtained from the Nagasaki University Hospital IRB (#23082107). Because this was a retrospective study, patient-identifiable information was removed and the study design was posted on the Nagasaki University Hospital's website along with an opt-out option, as directed by the IRB.

## Results

Histological study

This study included 43 patients with osteoporosis who underwent surgery. Because few patients with malignant tumors were treated with BP and most were treated with DMB, histological investigation was conducted only in patients with osteoporosis. The average age of the patients was 81.5 ± 8.81 years, with six men and 27 women.

**Figure 4:**
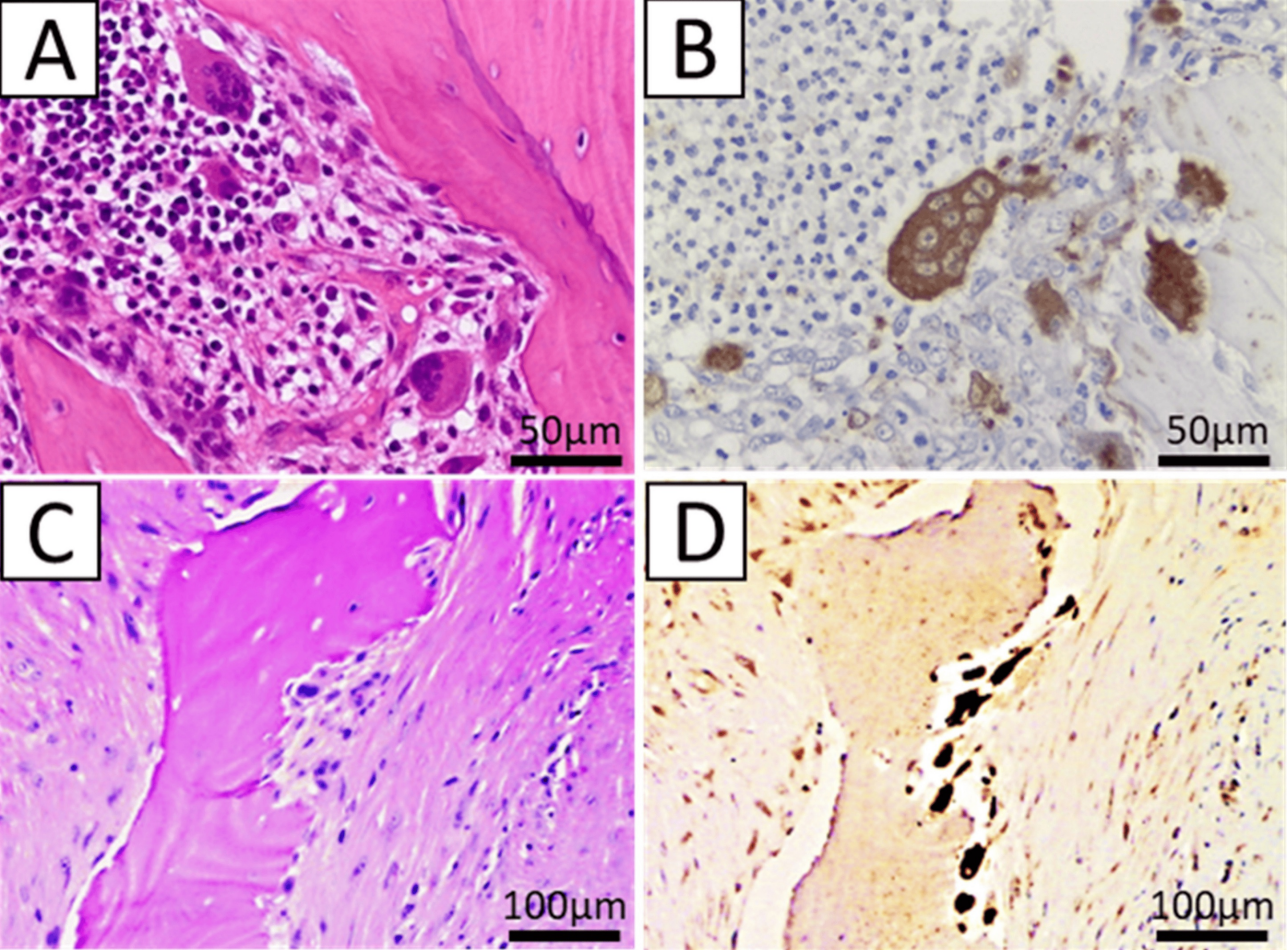
Histologic features. Large osteoclasts with numerous nuclei detached from the bone surface in patients with BP-related ONJ. (A) Hematoxylin-eosin and (B) cathepsin K staining. Small mononuclear or small multinucleated cells on the bone surface in patients with DMB-related ONJ. (C) Hematoxylin-eosin and (D) cathepsin K staining. BP: bisphosphonate; ONJ: osteonecrosis of the jaw; DMB: denosumab.

Among the 30 patients who received BP, 25 exhibited the detachment of large osteoclasts with a high number of nuclei from the bone surface. The degree of osteoclast suppression was mild in two, moderate in 11, severe in 14, and unknown in three patients. In contrast, small fractions of cathepsin-positive cells were found on the bone surface in 12 of 13 patients who received DMB (Figure 4). The degree of osteoclast suppression was mild in none, moderate in two, severe in 10, and unknown in one patient (Table 2). Thus, DMB exhibited more pronounced histological osteoclast suppression in many cases than BP.

**Table 2:** Degree of osteoclast suppression by antiresorptive agent.

Type of antiresorptive agent	Normal osteoclast	Suppressed osteoclast	Degree of suppression	Number of patients
Bisphosphonate	+	-	Mild	2
	+	+	Moderate	11
	-	+	Severe	14
	-	-	Unknown	3
Bisphosphonate→Denosumab	+	-	Mild	0
	+	+	Moderate	1
	-	+	Severe	7
	-	-	Unknown	0
Denosumab	+	-	Mild	0
	+	+	Moderate	1
	-	+	Severe	3
	-	-	Unknown	1

Serum bone metabolism markers, bone mineral density, and local bone metabolism markers

A total of 23 patients were enrolled in this study. Among them, BP was prescribed for three men and 15 women, with a mean age of 78.1 ± 8.6 years. Of these patients, 14 had osteoporosis, and four had malignant tumors. In contrast, DMB was administered to four men and one woman, with an average age of 68.6 ± 11.6 years. Among them, two patients had osteoporosis, and three had malignant tumors.

**Figure 5:**
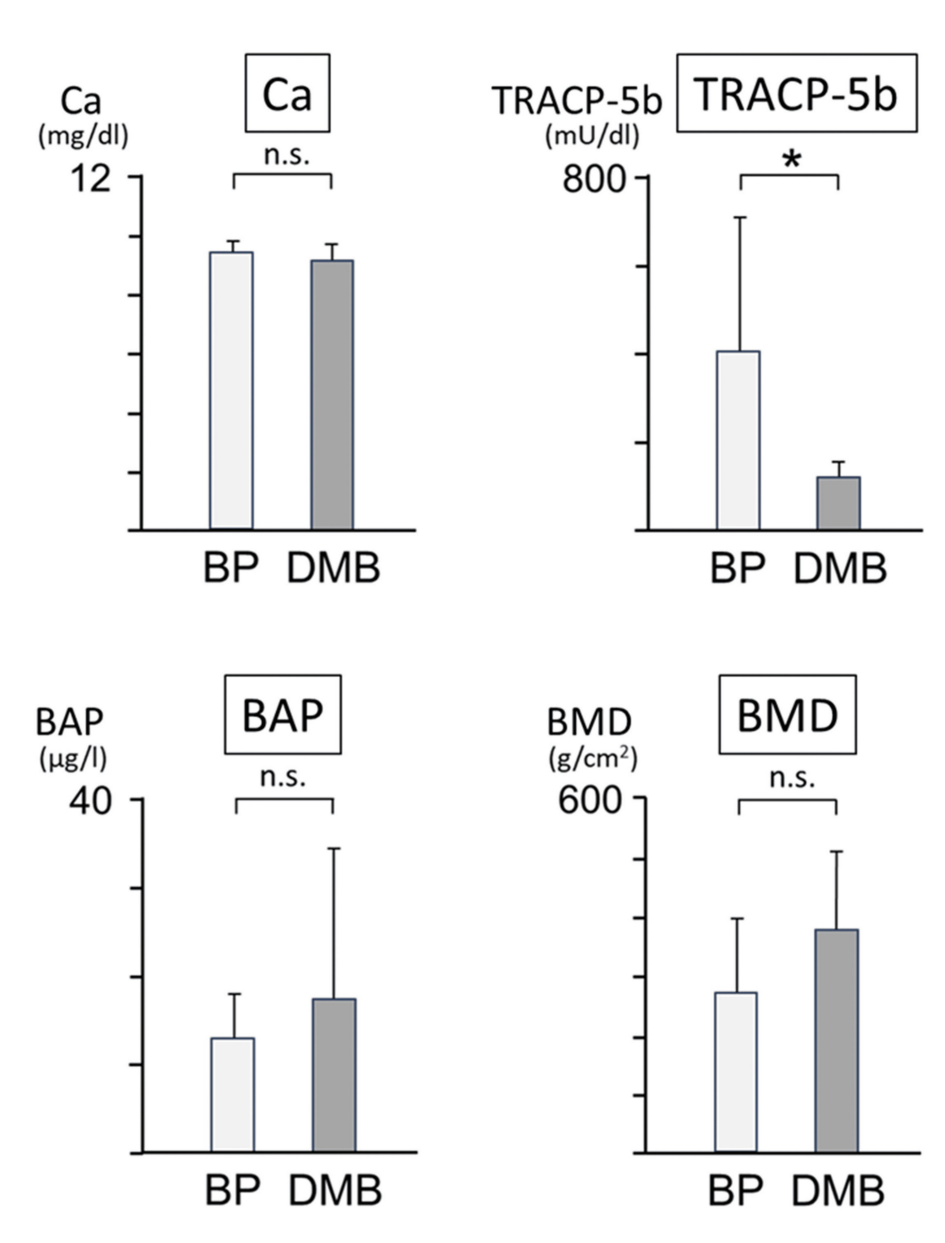
Serum bone metabolism markers (Ca, TRACP-5b, and BAP) and bone mineral density (BMD). Ca: calcium; TRACP-5b: tartrate-resistant acid phosphatase 5b; BAP: bone alkaline phosphatase; BP: bisphosphonate; DMB: denosumab; *: significant; n.s.: not significant.

No significant changes were observed in serum Ca and BAP levels among patients receiving BP and DMB. However, the serum TRACP-5b levels in the DMB group were significantly lower than those in the BP group. BMD was measured to evaluate bony characteristics. BMD values at the third cervical vertebra were not significantly different between the BP and DMB groups. These results indicated that although serum TRACP-5b levels were low in the DMB group, the BMD values did not differ between the two groups (Figure 5).

**Figure 6:**
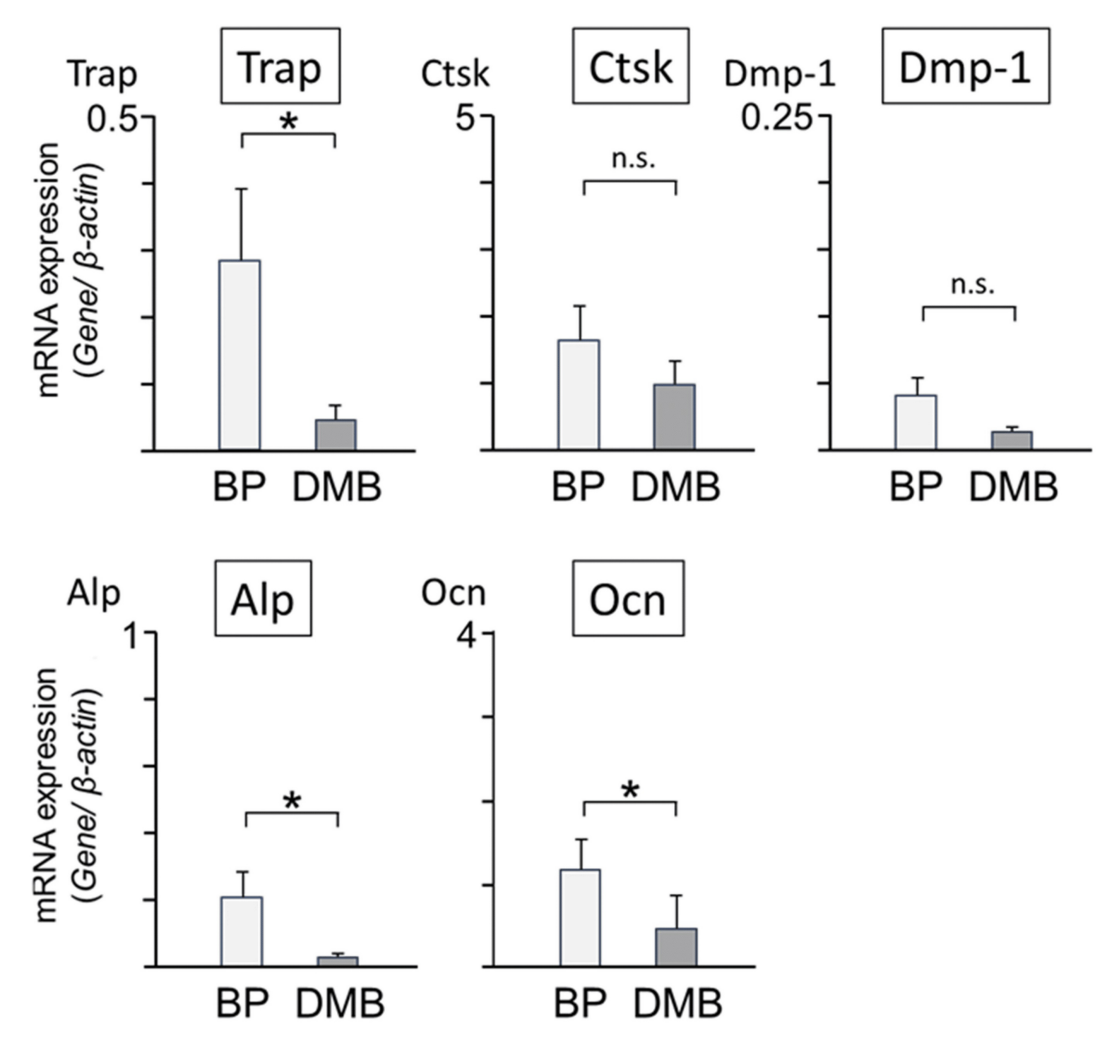
Bone metabolism-related genes in the bone (TRAP, CTSK, DMP-1, ALP, and OCN). TRAP: tartrate-resistant acid phosphatase; CTSK: cathepsin K; DMP-1: dentin matrix acidic phosphoprotein-1; BP: bisphosphonate; DMB: denosumab; ALP: alkaline phosphatase; OCN: osteocalcin; *: significant; n.s.: not significant.

Expression levels of genes related to bone metabolism were also evaluated. Figure 6 summarizes the comparison among the three groups regarding the expression levels of osteoclast-, osteocyte-, and osteoblast-related mRNAs (tartrate-resistant acid phosphatase (TRAP), cathepsin K (CTSK), dentin matrix acidic phosphoprotein-1 (DMP-1), alkaline phosphatase (ALP), and osteocalcin (OCN)). The expression level of TRAP in the DMB group was significantly lower than that in the BP group. The expression levels of CTSK and DMP-1 showed no significance between the two groups. The mRNA levels of ALP and OCN in the DMB group were significantly lower than those in the BP group. These results suggested that patients receiving DMB showed lower bone metabolic turnover than those receiving BP.

Clinical study

Of the 298 patients, 69 were men and 229 were women, with a mean age of 76.3 ± 11.1 years. BP was used in 187 patients, DMB in 85 patients, and BP was changed to DMB in 33 patients (Table 3).

**Table 3:** Characteristics of patients in clinical studies.

Variable	Category	Number of patients/mean ± standard deviation
Sex	Female	229
	Male	69
Age		76.3 ± 11.1
Medication-related osteonecrosis of the jaw site	Upper jaw	97
	Lower jaw	201
Medication-related osteonecrosis of the jaw stage	Stage 0	16
	Stage 1	21
	Stage 2	201
	Stage 3	60
Primary disease	Osteoporosis	187
	Malignancy	111
Type of antiresorptive agent	Bisphosphonate	180
	Denosumab	85
	Bisphosphonate→Denosumab	33
Duration of antiresorptive agent administration		55.1 ± 39.0
Drug holiday > 2 months	(-)	244
	(+)	51
Body mass index		21.1 ± 3.95
Neutrophil	×1,000/μL	4.45 ± 1.91
Lymphocyte	×1,000/μL	1.60 ± 0.753
Neutrophil-to-lymphocyte ratio		3.43 ± 2.53
Number of surgeries	1	245
	2	41
	3	12
Bone exposure	(-)	147
	(+)	151
Fistula reaching the bone	(-)	131
	(+)	167
Separation of sequestrum	(-)	172
	(+)	126
Degree of osteolysis	(-)	25
	Localized	161
	Extended	112
Periosteal reaction	(-)	213
	Attached-type	59
	Gap-type	21
	Irregular-type	5
Mixed-type osteosclerosis	(-)	275
	(+)	23
Total		298

**Figure 7:**
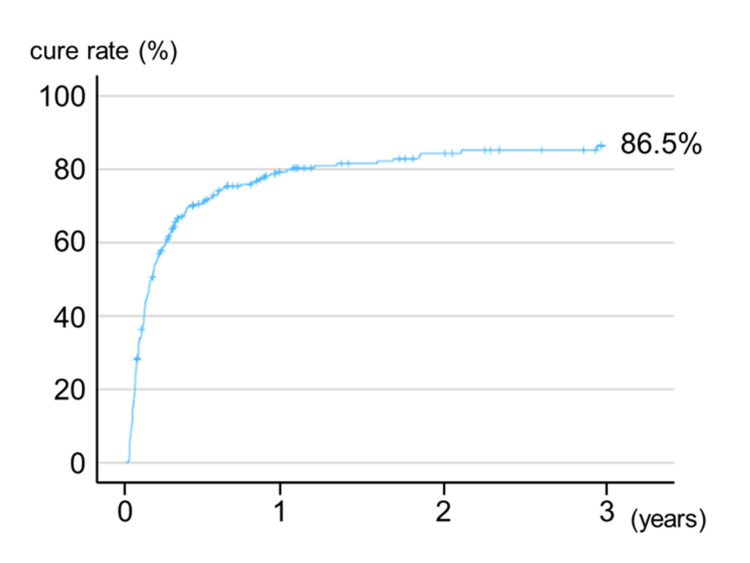
The cure rate of patients with medication-related osteonecrosis of the jaw.

First, the prognosis was examined. The three-year cure rate for all 298 patients was 86.5% (Figure 7). As factors related to treatment outcome, univariate analysis showed poor prognosis in men, younger patients, mandibular origin, malignancy, exposed bone, no osteolysis, irregular and pore-type periosteal reaction, and mixed osteosclerosis. Multivariate analysis, in which the ARA type was added as a covariate to the factors that were significant in the univariate analysis, showed that malignancy, gap-type or irregular-type periosteal reactions, and mixed-type osteosclerosis were significantly associated with a poor treatment course. Moreover, although the difference was not statistically significant, non-osteolytic MRONJ (p = 0.051) and those receiving DMB (p = 0.091) tended to have poorer treatment outcomes (Table 4).

**Table 4:** Factors related to the treatment outcome. * p < 0.05.

Variable	Category	Univariate analysis				Multivariate analysis		
		p-value	Hazard ratio	95% confidence interval		p-value	Hazard ratio	95% confidence interval
Sex	Male/female	0.034*	0.703	0.508-0.974		0.769	0.948	0.663-1.356
Age		0.112	1.010	0.998-1.022		-	-	-
Medication-related osteonecrosis of the jaw site	Lower/upper jaw	0.017*	0.720	0.550-0.943		0.067	0.757	0.562-1.020
Medication-related osteonecrosis of the jaw stage	Stage 3/2/1/0	0.349	1.091	0.909-1.309		-	-	-
Primary disease	Malignancy/osteoporosis	<0.001*	0.521	0.393-0.692		0.012*	0.617	0.424-0.898
Type of antiresorptive agent	Denosumab or both/bisphosphonate	0.255	0.857	0.658-1.117		0.097	1.291	0.955-1.744
Duration of antiresorptive agent administration (months)		0.883	1.000	0.996-1.003		-	-	-
Drug holiday > 2 months	(+)/(-)	0.799	1.046	0.739-1.482		-	-	-
Body mass index		0.286	0.982	0.951-1.015		-	-	-
Albumin		0.472	0.918	0.728-1.158		-	-	-
Neutrophil-to-lymphocyte ratio		0.874	0.996	0.944-1.050		-	-	-
Bone exposure	(+)/(-)	0.024*	0.744	0.575-0.963		0.056	0.758	0.571-1.007
Fistula reaching the bone	(+)/(-)	0.321	1.141	0.880-1.479		-	-	-
Separation of sequestrum	(+)/(-)	0.014*	1.381	1.067-1.786		0.838	1.030	0.775-1.370
Osteolysis	(+)/(-)	0.012*	2.100	1.174-3.757		0.051	1.875	0.997-3.527
Degree of osteolysis	Extended/localized/(-)	0.948	1.007	0.821-1.235		-	-	-
Periosteal reaction	Irregular or gap/attached/(-)	0.002*	0.713	0.576-0.883		0.019*	0.759	0.602-0.956
Mixed-type osteosclerosis	(+)/(-)	<0.001*	0.279	0.143-0.545		0.037*	0.468	0.229-0.957

Furthermore, we examined the differences between BP- and DMB-related ONJ. The patients who switched from BP to DMB were included in the DMB group. Univariate analysis revealed significant differences regarding sex, age, primary disease, ARA administration duration, serum albumin level, sequestrum separation, and osteolysis. Multivariate analysis showed that DMB-related ONJ was significantly more common in patients with malignant tumors, with a short duration of ARA administration, low serum albumin levels, and no osteolysis (Table 5). Additionally, univariate analysis of factors related to osteolysis revealed that sex, age, primary disease, ARA type, ARA administration duration, serum albumin level, sequestrum separation, and periosteal reaction were extracted. Multivariate analysis showed that non-osteolytic MRONJ was more common in patients with malignant tumors undergoing DMB therapy and less common in patients with periosteal reactions (Table 6).

**Table 5:** Comparison between bisphosphonate- and denosumab-related osteonecrosis of the jaw. * p < 0.05.

Variable		Univariate analysis				Multivariate analysis		
		Bisphosphonate-related medication-related osteonecrosis of the jaw	Denosumab-related medication-related osteonecrosis of the jaw	p-value		p-value	Odds ratio	95% confidence interval
Sex	Male	34	35	0.036*		0.396	0.721	0.339-1.534
	Female	146	83					
Age		78.0 ± 10.5	73.7 ± 11.4	<0.001*		0.663	0.993	0.962-1.025
Medication-related osteonecrosis of the jaw site	Upper jaw	56	41	0.529		-	-	-
	Lower jaw	124	77					
Medication-related osteonecrosis of the jaw stage	Stage 0	9	7	0.449		-	-	-
	Stage 1	14	7					
	Stage 2	115	85					
	Stage 3	41	19					
Primary disease	Osteoporosis	143	44	<0.001*		0.002*	3.508	1.600-7.693
	Malignancy	37	74					
Duration of antiresorptive agent administration		64.7 ± 41.4	41.2 ± 30.5	<0.001*		0.008*	0.987	0.978-0.997
Drug holiday > 2 months	(-)	148	96	0.875		-	-	-
	(+)	30	21					
Body mass index		20.9 ± 3.77	21.5 ± 4.20	0.214		-	-	-
Neutrophil		4.36 ± 1.60	4.57 ± 2.29	0.389		-	-	-
Lymphocyte		1.65 ± 0.653	1.54 ± 0.753	0.207		-	-	-
Neutrophil-to-lymphocyte ratio		3.22 ± 2.16	3.76 ± 2.98	0.080		-	-	-
Albumin		3.67 ± 0.561	3.51 ± 0.601	0.021*		0.013*	0.494	0.283-0.863
Number of surgeries	1	148	97	0.500		-	-	-
	2	23	18					
	3	9	3					
Bone exposure	(-)	93	54	0.344		-	-	-
	(+)	87	64					
Fistula reaching the bone	(-)	72	59	0.096		-	-	-
	(+)	108	59					
Separation of sequestrum	(-)	89	83	<0.001*		0.155	0.620	0.321-1.198
	(+)	91	35					
Degree of osteolysis	(-)	1	24	<0.001*		0.010*	0.065	0.008-0.518
	Localized	100	61					
	Extended	79	33					
Periosteal reaction	(-)	128	85	0.075		-	-	-
	Attached-type	41	18					
	Gap-type	10	11					
	Irregular-type	1	4					
Mixed-type osteosclerosis	(-)	169	106	0.267		-	-	-
	(+)	11	12					

**Table 6:** Characteristics of non-osteolytic medication-related osteonecrosis of the jaw. * p < 0.05.

Variable		Univariate analysis				Multivariate analysis		
		Non-osteolytic medication-related osteonecrosis of the jaw	Osteolytic medication-related osteonecrosis of the jaw	p-value		p-value	Odds ratio	95% confidence interval
Sex	Male	10	59	0.047*		0.508	1.427	0.498-4.088
	Female	15	214					
Age		71.1 ± 8.15	76.8 ± 11.2	0.014*		0.396	0.978	0.928-1.030
Medication-related osteonecrosis of the jaw site	Upper jaw	9	88	0.825		-	-	-
	Lower jaw	16	185					
Medication-related osteonecrosis of the jaw stage	Stage 0	2	14	0.072		-	-	-
	Stage 1	2	19					
	Stage 2	21	180					
	Stage 3	0	60					
Primary disease	Osteoporosis	1	186	<0.001*		0.003*	0.038	0.004-0.336
	Malignancy	24	87					
Type of antiresorptive agent	Bisphosphonate	1	179	<0.001*		0.003*	0.038	0.004-0.323
	Denosumab	16	69					
	Bisphosphonate→ Denosumab	8	25					
Duration of antiresorptive agent administration		38.4 ± 36.5	56.9 ± 39.0	0.025*		0.508	0.994	0.977-1.011
Drug holiday > 2 months	(-)	22	222	0.589		-	-	-
	(+)	3	48					
Body mass index		21.5 ± 3.87	21.1 ± 3.97	0.633		-	-	-
Neutrophil		4.55 ± 2.16	4.44 ± 1.88	0.770		-	-	-
Lymphocyte		1.86 ± 1.42	1.58 ± 0.650	0.075		-	-	-
Neutrophil-to-lymphocyte ratio		3.26 ± 2.44	3.46 ± 2.55	0.717		-	-	-
Albumin		3.54 ± 0.605	3.61 ± 0.581	0.550		-	-	-
Bone exposure	(-)	10	137	0.405		-	-	-
	(+)	15	136					
Fistula reaching the bone	(-)	14	117	0.214		-	-	-
	(+)	11	156					
Separation of sequestrum	(-)	22	150	0.001*		0.351	1.967	0.475-8.147
	(+)	3	123					
Periosteal reaction	(-)	24	189	0.036*		0.012*	4.836	1.410-16.589
	Attached-type	0	59					
	Gap-type	1	20					
	Irregular-type	0	5					
Mixed-type osteosclerosis	(-)	22	253	0.424		-	-	-
	(+)	3	20					

## Discussion

This study showed that local bone metabolism is more strongly suppressed in DMB-related ONJ than in BP-associated ONJ. Clinically, DMB-related ONJ is more common without osteolysis and tends to have a poorer treatment outcome.

Regarding the histopathology of MRONJ, specific findings in the osteonecrosis area have not been reported compared to typical bacterial osteomyelitis or ORN. Weinstein et al. [25] and Cho et al. [26] reported that the osteoclasts in patients treated with BP were megakaryocytes, multinucleated cells, and giant cells free from the bone surface. Our findings also indicated the presence of large osteoclasts with numerous nuclei detached from the bone surface in patients who had received BP treatment. In contrast, Miller [11] reported the almost complete disappearance of osteoclasts in mice; however, such reports in humans are scarce. Matsushita et al. [9] reported that in patients treated with DMB, cathepsin K-positive cells with very few nuclei existed or were detached from the bone surface. In this study, immature mononuclear cells and a few multinucleated cells were observed in patients treated with DMB, and typical multinucleated osteoclasts were rarely observed, aligning with findings reported by Matsushita et al. [9].

The association between markers of bone metabolism in blood and the development of MRONJ has not yet been established. Our results showed that the levels of RACP-5b, a marker of osteoclasts, decreased in the DMB group; however, no difference was observed between BP and DMB regarding Ca, BMD, and BAP levels. Therefore, we examined bone metabolism markers in the bone tissue rather than in the blood. The results of the search for bone metabolism-related genes in the bone tissue showed that the expression of TRAP, CTSK, DMP-1, ALP, and OCN tended to decrease in patients treated with DMB, with significant differences observed, especially for TRAP, ALP, and OCN. This suggests that DMB suppresses bone resorption as well as bone formation and strongly impairs bone remodeling as a whole; however, the number of cases studied was small, making it difficult to draw clear conclusions.

In clinical studies, no reports examined the differences between DMB- and BP-related ONJ. Recently, a preference for utilizing DMB over BP has emerged in patients with malignant tumors. Multivariate analysis demonstrated that a significantly higher proportion of patients treated with DMB exhibited no bone resorption than those treated with BP. As factors related to prognosis, multivariate analysis showed that malignancy as a primary disease, gap-/irregular-type periosteal reaction, and mixed-type osteosclerosis were significantly associated with poor treatment outcomes. Patients with non-osteolytic disease and those receiving DMB also tended to have a poorer prognosis, although this difference was not statistically significant. Although drawing a definitive conclusion is challenging owing to the limited number of cases, the findings suggest that bone resorption was not consistently observed in patients treated with DMB. This complexity in assessing bone involvement during surgery may have contributed to increased postoperative recurrences. In the future, we intend to use magnetic resonance imaging and single-photon emission CT to investigate methods for determining the appropriate range of bone resection in non-osteolytic MRONJ.

This study had some limitations. As this was a retrospective study conducted at a single institution with a small number of cases, generalizing the obtained results is difficult. Moreover, many patients with malignant tumors received DMB rather than BP, making it challenging to differentiate between the two medications in this study when the primary diseases were matched. However, to our knowledge, this is the first study to show that DMB has a stronger inhibitory effect on local bone metabolism than BP and that patients treated with DMB tend to have more cases without osteolysis on imaging and a poorer treatment outcome. In the future, we would like to increase the number of cases to clarify the differences in pathophysiology and clinical characteristics between BP- and DMB-related ONJ and establish the optimal treatment.

## Conclusions

Histological examination of osteoclasts and real-time RT-PCR of bone metabolism-related genes suggested that DMB suppresses local bone metabolic turnover more strongly than BP.
