# Correction: Differences Between Medication-Related Osteonecrosis of the Jaw Caused by Bisphosphonates and Denosumab: Histological, Molecular Biological, and Clinical Studies

**DOI:** 10.7759/cureus.c210

**Published:** 2025-02-04

**Authors:** Taro Miyoshi, Mitsunobu Otsuru, Kota Morishita, Keisuke Omori, Kei-ichiro Miura, Saki Hayashida, Satoshi Rokutanda, Yuki Matsushita, Masahiro Umeda, Tomohiro Yamada

**Affiliations:** 1 Department of Oral and Maxillofacial Surgery, Nagasaki University Graduate School of Biomedical Sciences, Nagasaki, JPN; 2 Oral and Maxillofacial Surgery, Graduate School of Kanagawa Dental University, Kanagawa, JPN; 3 Oral and Maxillofacial Surgery, Nagasaki University, Nagasaki, JPN; 4 Dentistry and Oral Surgery, The Japanese Red Cross Nagasaki Genbaku Hospital, Nagasaki, JPN; 5 Dentistry and Oral and Maxillofacial Surgery, Juko Memorial Nagasaki Hospital, Nagasaki, JPN; 6 Cell Biology, Nagasaki University, Nagasaki, JPN

This article was originally published with the affiliation of the first author, Taro Miyoshi, missing part of its name. The affiliation's name has been corrected to “Department of Oral and Maxillofacial Surgery, Nagasaki University Graduate School of Biomedical Sciences”.

